# Delta-9-tetrahydrocannabinol alleviates inflammation by modulating immune responses in LPS-induced murine inflammation model

**DOI:** 10.3389/fimmu.2026.1856131

**Published:** 2026-09-16

**Authors:** Shallu Tomer, Jeffrey Harding, Li Wang, Nandita Kedia, Ethan Cook, Vincent Le, Christopher Platt, Jonathan Le, Nicholas Tien, Kanishkh Patalay, Peter Langfelder, X. William Yang, Ye Zhang, Anjie Zhen

**Affiliations:** 1 Division of Hematology and Oncology, David Geffen School of Medicine, University of California, Los Angeles, Los Angeles, CA, United States; 2 Department of Psychiatry and Biobehavioral Sciences, David Geffen School of Medicine, University of California, Los Angeles, Los Angeles, CA, United States; 3 Center for Neurobehavioral Genetics, Semel Institute for Neuroscience & Human Behavior, University of California, Los Angeles, Los Angeles, CA, United States

**Keywords:** cannabinoid, inflammation, LPS, neuroninflammation, THC - tetrahydrocannabinol

## Abstract

**Introduction:**

Δ^9^-Tetrahydrocannabinol (THC), the primary psychoactive component of cannabis, has been reported to modulate immune responses; however, its effects on complex innate immune signaling pathways and inflammation remain incompletely understood. Here, we investigated the immunomodulatory effects of THC on lipopolysaccharide (LPS)-induced inflammation across cellular and *in vivo* models.

**Methods:**

We integrated transcriptomic, cellular, and *in vivo* approaches to characterize the effects of THC on innate immune activation. Human THP-1 monocytic cells were treated with THC in the presence or absence of LPS, followed by transcriptomic and gene expression analyses to evaluate inflammatory, type I interferon, unfolded protein response (UPR), and autophagy pathways. Findings were validated in primary human monocytes by assessing immune activation markers. The effects of chronic THC exposure were further evaluated in a murine model of systemic inflammation by examining splenic myeloid and T-cell activation. RNA sequencing of brain tissue was performed to assess the effects of THC on neuroinflammatory and neuronal pathways.

**Results:**

THC induced a stress-adaptive transcriptional program in THP-1 cells, characterized by upregulation of genes associated with the UPR and autophagy pathways. In contrast, THC markedly suppressed LPS-induced type I interferon-stimulated genes (ISGs) and pro-inflammatory cytokines, including IL-1β and TNF-α. In primary human monocytes, THC significantly reduced the expression of activation markers CD80, CD83, and CD209. Consistent with these findings, chronic THC exposure attenuated the activation of splenic myeloid and T cells in mice subjected to systemic inflammation. Brain transcriptomic analysis further demonstrated reduced expression of neuroinflammatory pathways following THC exposure, accompanied by enrichment of pathways associated with neurogenesis and synaptic plasticity.

**Discussion:**

Our findings demonstrate that THC exerts broad immunomodulatory effects characterized by suppression of inflammatory and type I interferon responses while promoting cellular stress-adaptation pathways. These effects were observed across human monocytic cells, primary monocytes, and peripheral immune populations *in vivo*, and were accompanied by reduced neuroinflammatory signaling and increased neuronal plasticity-associated pathways in the brain. Together, these findings provide mechanistic insight into the immunomodulatory actions of THC and support further investigation of cannabinoid signaling as a potential regulator of chronic inflammatory and neuroimmune disorders.

## Introduction

Cannabis has been widely used for both medicinal and recreational purposes, with over 44.3 million individuals reporting marijuana consumption in the past month in the United States in 2024 (1). Its principal bioactive constituents, the psychoactive Δ^9^-tetrahydrocannabinol (THC) and the non-psychoactive cannabidiol (CBD), interact with the endocannabinoid system to exert immunomodulatory, anti-inflammatory, analgesic, antiemetic, and neuroprotective effects (2–9). Cannabinoid receptors are broadly distributed throughout the body (10). The effects of cannabinoids are primarily mediated by two G protein-coupled receptors (GPCRs), CB1 and CB2 (11). CB1 is primarily expressed in the central nervous system (CNS) and in peripheral tissues such as the liver, adipose tissue, and gastrointestinal tract, whereas CB2 is predominantly found in immune cells, including macrophages, T and B lymphocytes, and microglia, where it plays a critical role in regulating inflammatory responses (11–13).

Chronic inflammation is a central pathological feature of numerous diseases, including classical inflammatory disorders, metabolic and cardiovascular conditions, and neurodegenerative disorders such as Alzheimer’s and Parkinson’s disease (14–18). Lipopolysaccharide (LPS), a potent bacterial endotoxin, activates Toll-like receptor 4 (TLR4), initiating signaling cascades that induce pro-inflammatory cytokines such as TNF-α and IL-1β, leading to systemic and neuroinflammatory responses (19, 20). Owing to its ability to trigger robust immune activation, LPS serves as a well-established model for studying pathogen-driven inflammation, including systemic inflammation, sepsis, and neuroinflammation (21, 22).

Emerging evidence suggests that cannabinoids modulate innate immune signaling in both the brain and periphery, in part by targeting TLR4-mediated pathways critical for host defense and neuroinflammatory processes (23–25). Although multiple studies, including our own (26), have demonstrated the immunomodulatory properties of cannabinoids, their specific effects on LPS-induced inflammatory responses remain poorly defined (23–25, 27–29). Investigating the impact of cannabis-derived compounds, particularly THC, on LPS-driven immune activation may provide mechanistic insights into cannabinoid regulation of inflammatory and stress pathways, with potential implications for infection, neuroinflammation, and the development of novel therapeutic strategies.

In this study, we examined the effects of THC on LPS-induced inflammation in human THP-1 monocytic cells, primary human monocytes, and an *in vivo* mouse model of LPS-induced inflammation. We hypothesize that THC may attenuate LPS-induced inflammatory responses both systemically and in the CNS by reprogramming innate immune signaling pathways towards a stress-adaptive, less inflammatory state.

## Results

### RNA-seq analysis reveals differential gene expression following LPS stimulation in the presence and absence of THC in THP-1 cells

THC has been shown to exert anti-inflammatory effects by modulating immune cell function and attenuating innate immune signaling pathways (30). To gain a comprehensive understanding of the global transcriptional changes induced by THC in macrophages, we conducted an RNA sequencing (RNA-seq) analysis on THP-1 monocytic cells. Previously, we have shown that 10 µg/mL cannabidiol treatment led to down regulation of Type I interferon response genes (ISGs) in THP1 cells (26), and pilot real time PCR studies showed that 10ug/ml treatment of lipopolysaccharide (LPS) -stimulated THP-1 cell led to reduction of ISGs without cytotoxicity. Therefore, we performed RNAseq analysis using THP-1 cells that were treated with four distinct conditions for 8 hours: vehicle, THC (10ug/ml), LPS, and a combination of LPS and THC. Experiments were repeated in three biological replicates. This experimental setup allowed us to examine both the independent and combinatorial effects of THC on monocyte gene expression. Differential expression analysis with DESeq2 identified 7,832 DEGs in the THC versus vehicle comparison and 8,050 DEGs in the LPS + THC versus LPS comparison (adjusted p < 0.05, |log_2_FC| > 1). Our analysis revealed that THC treatment alone significantly impacted the expression of genes involved in various metabolic and autophagic pathways, as reported previously (31–33). Specifically, THC treatment upregulated genes related to Oxidative/Redox Stress & Ferroptosis Resistance (such as HMOX1, MT2A), Lipid Metabolism/PPARγ Activation (PPARG, ACSL1), endoplasmic reticulum (ER) stress responses (such as HSPA5), autophagy and proteostasis (such as SQSTM1, MAP1LC3B) suggesting a broad effect on cellular homeostasis (34–37) (**Figure 1A**). Interestingly, when comparing THP-1 cells stimulated with LPS to those co-treated with both LPS and THC, we observed a marked downregulation of interferon-related genes, especially key Type I interferon (IFN) response genes (ISGs), such as chemokine CXCL10 and CXCL11, antiviral restriction factors APOBEC3G and APOBEC3A. These genes are crucial for antiviral immunity, indicating a potential immunomodulatory effect of THC in inflammatory conditions (38). Additionally, genes encoding DC-SIGN (CD209) and PD-L1 (CD274) were also suppressed in response to THC treatment in LPS stimulated THP-1 cells, both of which play important roles in immune signaling and inflammation (39, 40). Similar to upregulated genes observed in THC vs. mock treatment, we saw upregulation of genes associated with autophagy and ferroptosis in LPS and THC treatment as compared to LPS stimulation alone (**Figure 1B**). These changes suggest that THC modulates stress adaptation and metabolic pathways, accompanied by reduced expression of inflammatory genes to LPS stimulation in THP-1 cells.

**Figure 1:**
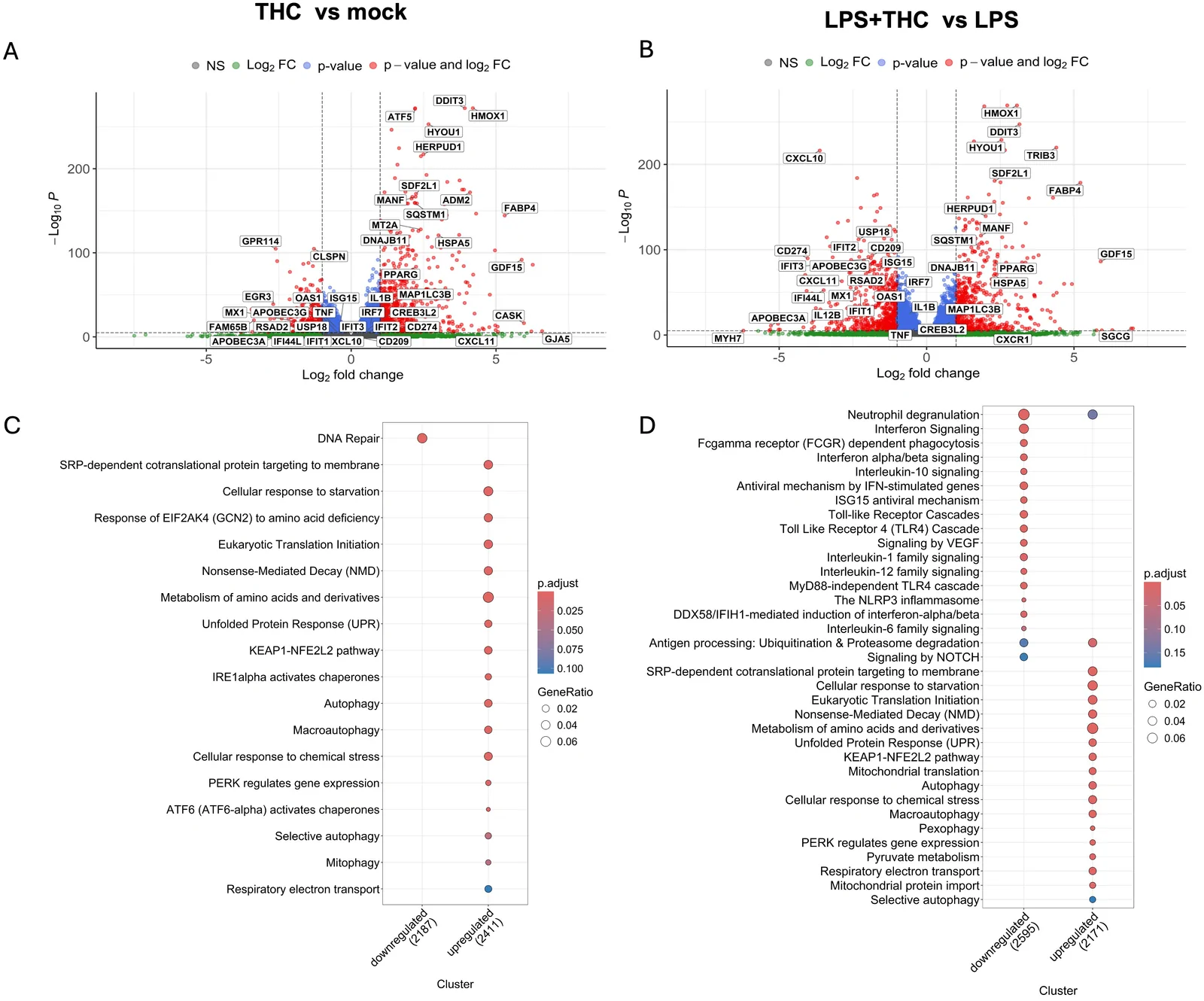
Differential gene analysis by RNA-seq in THC treated LPS-stimulated THP-1 cells. THP-1 monocytic cells were treated with vehicle and THC (10ug/mL) and stimulated in the presence and absence of LPS (5ug/mL). Cells were collected after 8hrs. Volcano Plots showing differential expression of genes in **(A)** Mock (n=3) vs THC (n=3) treated cells, **(B)** LPS+THC (n=3) vs LPS (n=3) treated THP-1 cells. Each point represents a single gene, plotted according to its log2 fold change (X-axis) and -log10 adjusted p-value (Bonferroni adjusted) (Y-axis). Significant differentially expressed genes (DEGs) are colored (red/blue) and top targets are labeled. **(C, D)** Dot plots of pathway enrichment analysis for **(C)** THC vs. Mock and **(D)** LPS+THC vs. LPS comparisons. Enriched pathways are stratified by regulation direction (downregulated vs. upregulated clusters). The size of the dots represents the GeneRatio (proportion of genes associated with the term), and the color gradient indicates statistical significance (adjusted p-value). Experiment was repeated three independent times and samples were submitted for RNA sequencing.

To systematically characterize the biological processes driving these transcriptional shifts, we performed Reactome pathway enrichment analysis stratified by differential regulation clusters. In the absence of inflammatory stimulation, THC treatment alone primarily enriched for pathways associated with cellular stress adaptation (**Figure 1C**). Specifically, upregulated genes were heavily clustered in the Unfolded Protein Response (UPR) (e.g., PERK regulates gene expression, IRE1alpha activates chaperones) and autophagy/macro-autophagy pathways. Additionally, we observed enrichment for the KEAP1-NFE2L2 pathway, reinforcing the role of THC in modulating oxidative stress responses.

Critically, this analysis highlighted a functional trade-off during inflammatory challenge. In LPS-stimulated cells, THC treatment resulted in a distinct bifurcation of pathway activity (**Figure 1D**). While metabolic and proteostatic stress pathways (including the UPR, mitochondrial translation, and amino acid metabolism) remained significantly upregulated, there was a profound suppression of innate immune signaling. The downregulated cluster was dominated by Type I interferon signaling, ISG15 antiviral mechanisms, and Toll-like receptor (TLR) cascades. Collectively, these data suggest that THC induces a conserved transcriptional program characterized by activation of ER stress, autophagy, and other stress-response pathways that persists during inflammatory challenge. This response is accompanied by reduced expression of type I interferon signaling, ISG15 antiviral pathways, and Toll-like receptor (TLR) signaling genes, consistent with a shift toward a less inflammatory transcriptional state.

To validate these RNA-seq findings, we performed quantitative PCR (qPCR) on a subset of differentially expressed genes. Specifically, we selected Type I IFN response genes (MX1, OAS1, IRF7) and pro-inflammatory cytokine genes (IL-1β, TNF-α) for further analysis. The qPCR results confirmed the downregulation of these genes in LPS-stimulated THP-1 cells treated with THC, compared to LPS-stimulated vehicle-treated controls (**Figure 2**). The fold changes observed in the qPCR analysis were consistent across biological replicates and aligned with the RNA-seq data, further reinforcing the validity of our transcriptomic findings. Statistical analyses of the qPCR data corroborated the significance of these expression changes, underscoring the robustness and reliability of our results.

**Figure 2:**
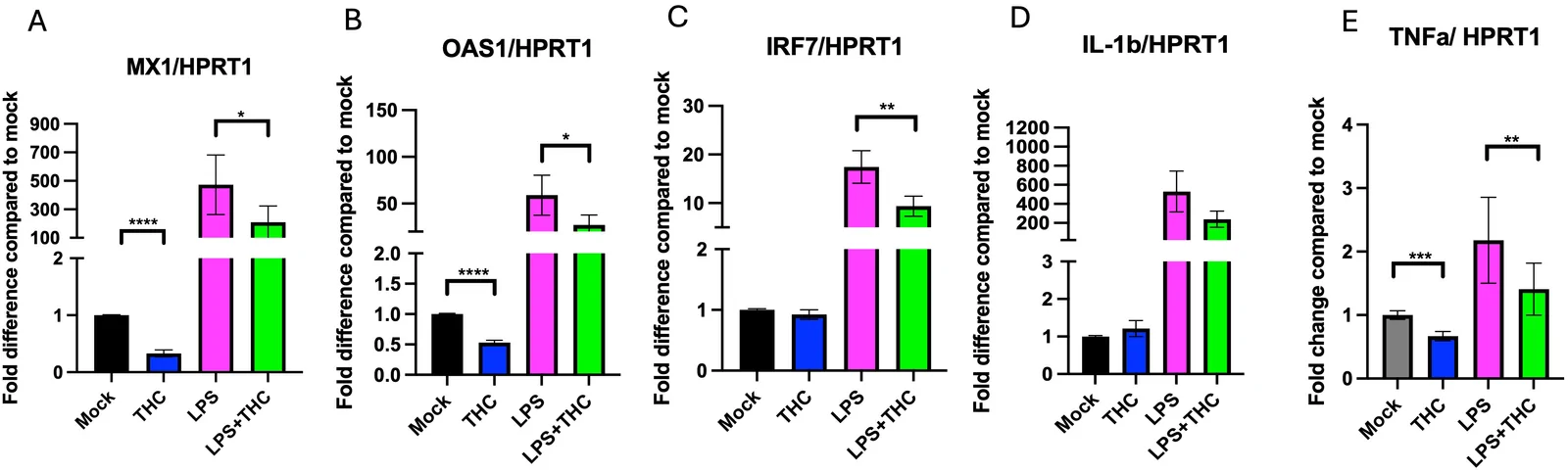
THC suppresses type I interferon and pro-inflammatory cytokine gene expression in LPS-stimulated THP-1 cells: THP-1 monocytic cells were treated with vehicle and THC (10ug/mL) and stimulated in the presence and absence of LPS (5ug/mL). Cells were collected after 8hrs from 3 independent experiments. Bar graphs showing the fold difference of gene expression as compared to mock for Type I IFN genes (MX1, OAS1, IRF7) and Pro-inflammatory genes (IL-1β, TNF-⍺) as **(A)** MX1, **(B)** OAS1 **(C)** IRF7, **(D)** IL-1β and, **(E)** TNF-⍺. *p<0.05, **p<0.005, ***p<0.0005. Data are presented as mean ± SEM.

Overall, our findings highlight the immunomodulatory effects of THC in monocytic THP-1 cells, demonstrating its capacity to suppress key antiviral and inflammatory responses while simultaneously influencing metabolic and cell death pathways.

### THC attenuates activation in primary monocytes

Heavy cannabis use has been associated with altered immune activation, underscoring its potential role as an immunomodulatory agent (41). Monocytes, when exposed to inflammatory stimuli such as lipopolysaccharide (LPS), exhibit an upregulation of activation markers, including CD80, CD83, CD209 (DC-SIGN), and MHC II (HLA-DR) (42). This upregulation signifies immune cell activation and the initiation of an inflammatory response when compared to unstimulated conditions (43).

To investigate the impact of THC on primary myeloid cells, we first examined the appropriate concentration dose for primary cells. An independent dose optimization study was performed using THC concentrations of 1 µg/mL, 2 µg/mL, 200 ng/mL, and 50 ng/mL. Cytotoxicity was observed following treatment with the higher THC concentrations (1 µg/mL and 2 µg/mL), whereas 200 ng/mL and 50 ng/mL THC were well tolerated without detectable cell death. Based on these findings, 200 ng/mL and 50 ng/mL THC concentrations were selected for subsequent experiments in primary cells. We treated primary PBMCs with LPS in presence of vehicle control or THC (50 ng/mL and 200 ng/mL) for eight days. Flow cytometric analysis showed that THC significantly reduced CD80, CD83, CD209 expression in LPS-stimulated, THC-treated CD11b+ cells compared to LPS-treatment only controls (**Figure 3A**.). As shown in **Figure 3B**, we observed consistently reduced expression of activation markers by THC in the presence of LPS across four different donors (**Figure 3B**), suggesting THC’s role in dampening myeloid cells activation by LPS. Interestingly, we did not observe changes in LPS mediated T cell activation by THC (**Supplementary Figure 1**). Gating strategy for CD11b+ cells (**Figure 3**) and CD4+ and CD8+ cells (**Supplementary Figure 1**) is represented in **Supplementary Figure 2**.

**Figure 3:**
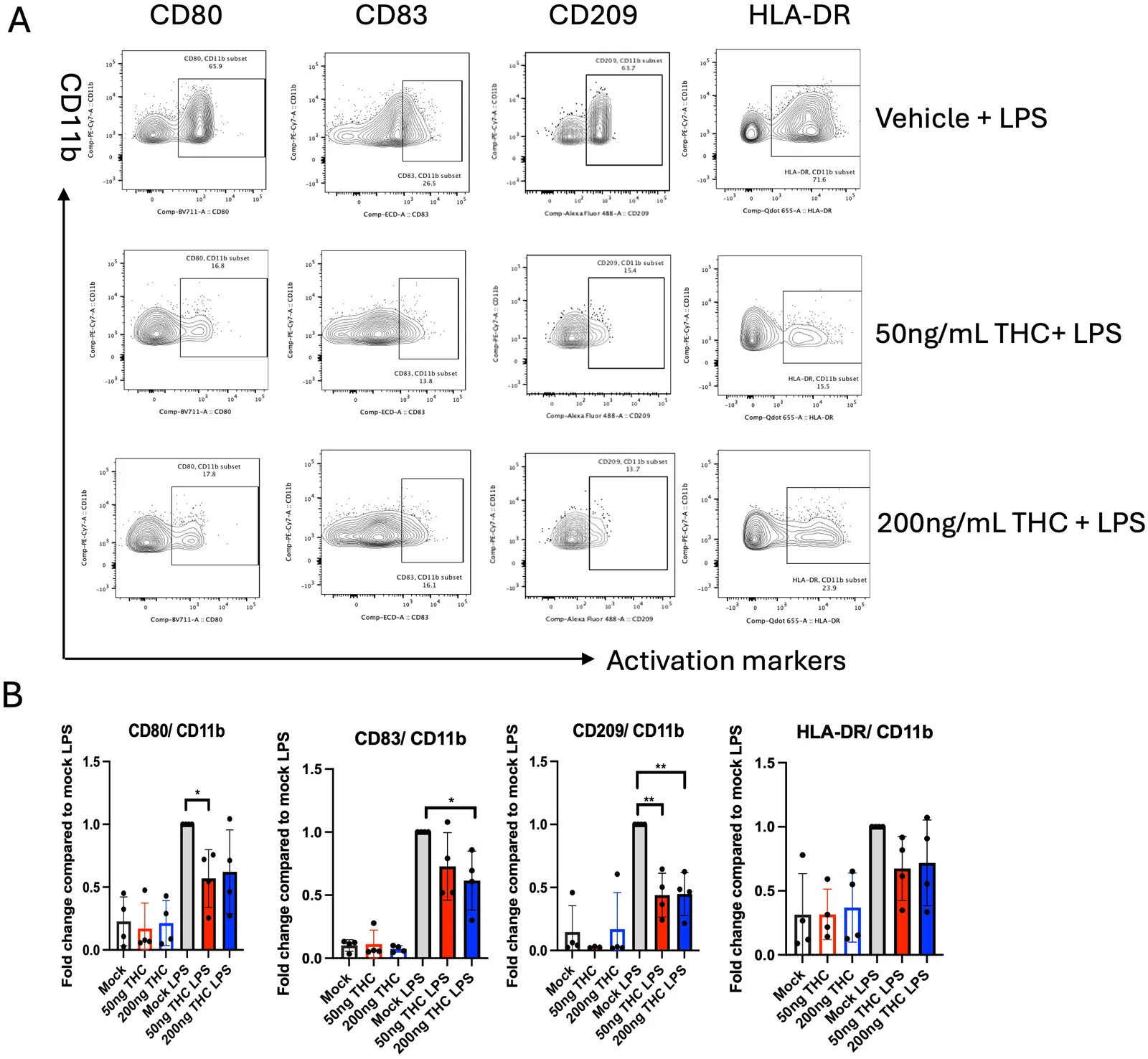
THC reduces the expression of activation markers in primary monocytes: PBMCs were treated with vehicle and THC (50ng/mL, 200ng/mL) in the presence and absence of LPS (5ug/mL) for 8 days. Cells were harvested and stained for activation markers CD80, CD83, CD209 and HLA-DR. **(A)** Representative flow plots for activation markers, **(B)** Summary of fold change compared to mock and LPS in terms of percentage of cells positive for activation markers from 4 healthy donors. *p<0.05, **p<0.005.

### Chronic THC exposure reduces LPS-induced immune activation *in vivo*

To investigate whether THC modulates *in vivo* immune activation, we designed a controlled study utilizing wild-type immunocompetent B6/129J mice. Animals were randomly assigned to receive either a control diet or a THC-supplemented diet (20 mg/kg/day) for 15 consecutive days. The THC dose used in the study was selected based on previously published studies demonstrating tolerability and biological activity of THC at similar doses in mice (32, 44). Beginning on day 12, mice were challenged intraperitoneally (i.p.) with either vehicle or LPS (0.5 mg/kg) once daily for three consecutive days to induce a state of systemic inflammation and mimic pathogen-associated immune activation. On day 15, mice were euthanized, and multiple tissues (blood, spleen, and brain) were harvested for immunological and biochemical analyses (**Figure 4A**).

**Figure 4:**
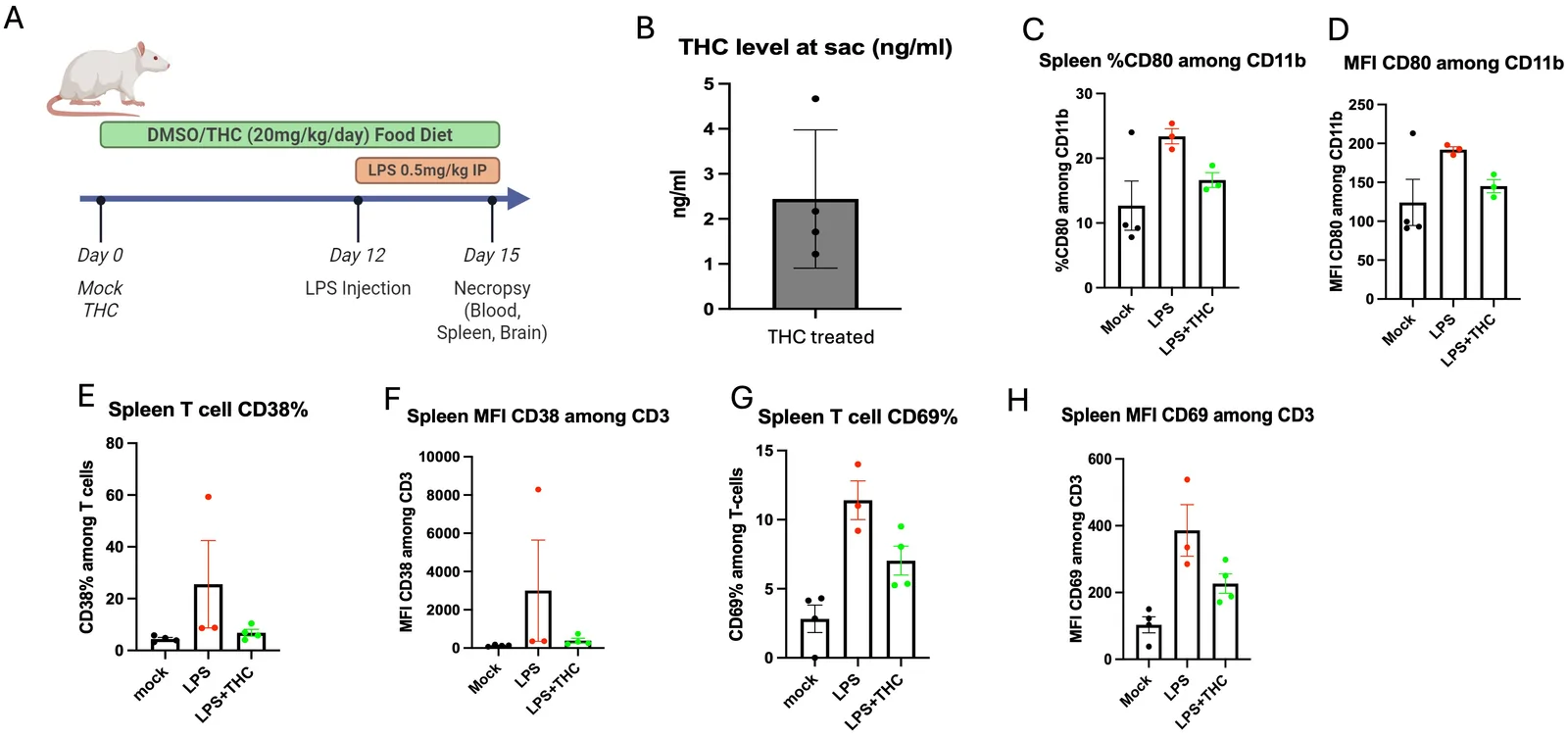
THC reduces LPS-induced immune activation *in vivo*. Wild-type mice were fed with either control diet or diet supplemented with THC (20mg/kg/day) for 15 consecutive days. At day 12, mice were injected control or 0.5mg/kg LPS by IP injection for 3 days to induce inflammation. **(A)** Schematic representation of mice groups and THC/LPS treatment regime. **(B)** Plasma THC levels measured by HPLC in THC-treated mice. Flow cytometric analysis showed reduced expression of the activation marker in THC treated mice. **(C)** Percentage of cells positive for CD80 among CD11b in spleen, **(D)** MFI of cells positive for CD80 among CD11b in spleen. **(E)** Percentage of cells positive for CD38 on CD3^+^ T cells in spleen, **(F)** MFI of cells positive for CD38 on CD3^+^ T cells in spleen and **(G)** Percentage of cells positive for CD69 on CD3^+^ T cells on spleen, **(H)** MFI of cells positive for CD69 on CD3^+^ T cells in spleen.

To confirm systemic THC bioavailability, plasma samples collected at necropsy were analyzed using high-performance liquid chromatography (HPLC). THC-treated animals demonstrated a mean plasma concentration of 2.44 ng/mL, confirming consistent *in vivo* exposure across the treatment period (**Figure 4B**). Notably, these THC levels are below the ‘legally impaired’ blood concentration of THC (5ng/mL) used in Illinois, Montana and Washington (45). Although this legal threshold is not directly applicable to mice and should not be interpreted as a biological threshold for intoxication, comparison with reported human plasma THC concentrations provides translational context, indicating that our dosing regimen achieved systemic THC exposure within a clinically relevant range without producing markedly elevated circulating THC levels.

Flow cytometric profiling of splenocytes revealed that while LPS induce elevated expression of CD80, THC treatment markedly reduced CD80 expression on splenic CD11b^+^ myeloid cells (**Figures 4C, D**). Interestingly, while *in vitro*, short term THC treatment had no effect on T cells (**Supplementary Figure 1**), THC administration attenuated the expression of canonical activation markers CD38 and CD69 on CD3^+^ T cells relative to vehicle-treated controls, suggesting suppression of peripheral T cell activation (**Figures 4E–H**). Gating strategy for CD11b+CD80+ dual positive and CD3+CD38+/CD3+CD69+ dual positive cells is represented in **Supplementary Figure 3**.

### THC attenuates LPS-induced neuroinflammation in brain tissue

While CB2 receptor is expressed on immune cells, the main cannabis receptor CB1 is expressed mainly in the central nervous system (CNS) (46). To investigate whether the immunomodulatory effects of THC extend to the CNS, we performed bulk RNA sequencing analysis on brain tissue from mice. Brain samples were collected from mice treated with control diet (n=3), LPS alone (n=3), or THC along with LPS (n=3), allowing us to examine both LPS-induced neuroinflammatory responses and THC’s modulatory effects on these processes.

Comparison of LPS-treated versus mock-treated brain tissue revealed a robust inflammatory response characterized by significant upregulation of numerous genes involved in immune activation and inflammatory signaling (**Figure 5A**). Differential expression analysis with DESeq2 identified 1876 DEGs (adjusted p < 0.05, |log_2_FC| > 1), shown in the volcano plot in **Figure 5A** and 504 DEGs shown in **Figure 5B**. Among the most prominently upregulated genes were classical inflammatory markers and cytokines, consistent with LPS-induced neuroinflammation.

**Figure 5:**
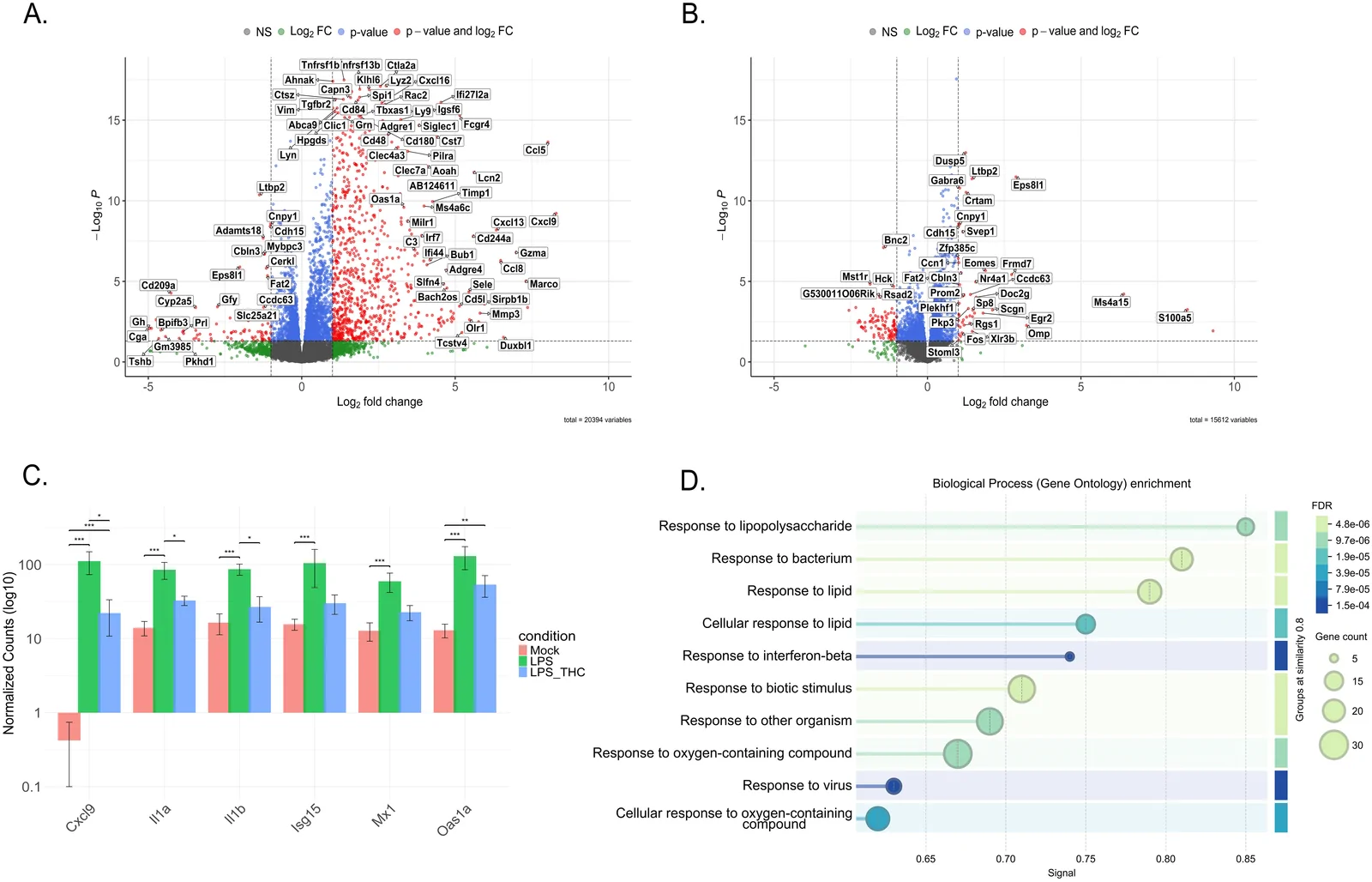
RNA sequencing analysis of brain tissue reveals differential gene expression patterns in response to LPS and THC treatment. **(A)** Volcano plot comparing LPS (n=3) vs Mock (n=3) treatment conditions. X-axis represents log2 fold change in gene expression; Y-axis represents –log10 p-value (Bonferroni adjusted). Green points indicate genes with non-significant changes (NS), blue points represent genes with significant log2 fold change (Log2FC), and red points indicate genes meeting both p-value and log2 fold change significance thresholds (p-value and log2FC). Dashed lines indicate significance cutoffs. **(B)** Volcano plot comparing LPS+THC (n=3) vs LPS (n=3) treatment conditions using the same color scheme and significance thresholds as panel **(A)** Selected genes of interest are labeled. **(C)** Expression levels of selected genes across treatment conditions. Bar graph shows normalized counts (log10 scale) for six representative genes (Cxcl9, IL-1⍺, IL-1β, Isg15, Mx1, and Oas1a across three conditions: Mock (red), LPS (green), and LPS+THC (blue). Error bars represent standard error of the mean. Statistical significance between groups is indicated by asterisks (*p < 0.05, **p < 0.01, ***p < 0.001). **(D)** Gene Ontology (GO) biological process enrichment analysis for downregulated genes in LPS+THC vs LPS comparison. Dot plot shows significantly enriched biological processes with signal strength and statistical significance indicated by color intensity. Circle size represents gene count. Enriched processes include response to LPS, immune responses, cellular responses to interferon beta, and metabolic processes. A total of n=3 biological replicates per group were submitted for sequencing, including mock, LPS- treated, and LPS+THC- treated brain samples.

When comparing brain tissue from mice treated with LPS plus THC versus LPS alone, we observed a markedly different transcriptional profile (**Figure 5B**). THC co-treatment resulted in significant downregulation of numerous LPS-induced inflammatory genes (such as Cxcl9, Il1b, Il1a, Cx3cr1, Lbp, Ly86, Cd180), suggesting that THC effectively dampens neuroinflammatory responses even after inflammatory stimuli have been introduced. Several key inflammatory mediators and immune response genes showed reduced expression in the presence of THC, indicating broad anti-inflammatory effects within the central nervous system. The upregulated genes are involved in neurogenesis, circadian rhythm regulation, and the modulation of synaptic plasticity and synaptic transmission (**Supplementary Figure 4**).

To validate these RNA-seq findings, we examined the expression patterns of six representative genes across all treatment conditions using quantitative analysis (**Figure 5C**). The selected genes, including Cxcl9 (immune cell migration), IL-1⍺ and IL-1β (pro-inflammatory cytokines), ISG15, MX1, and OAS1a (interferon-stimulated gene, demonstrated consistent patterns with our transcriptomic data. LPS treatment significantly upregulated expression of these inflammatory markers compared to mock controls, while THC co-treatment substantially reduced their expression levels, confirming the anti-inflammatory effects observed in the broader RNA-seq analysis.

Gene Ontology enrichment analysis of genes downregulated in the LPS plus THC versus LPS comparison revealed significant enrichment of biological processes central to immune function and inflammation (**Figure 5D**). The most significantly enriched pathways included response to lipopolysaccharide, response to bacterium, cellular response to lipid, immune response, and response to interferon-beta. Additional enriched processes encompassed response to biotic stimulus, response to other organisms, cellular response to lipid, and cell activation. The high statistical significance and substantial gene counts within these pathways underscore the coordinated suppression of neuroinflammatory processes by THC treatment. Together, these findings suggest that THC modulates immune cell transcriptional programs associated with inflammatory responses, supporting its immunomodulatory effects in the context of LPS-induced inflammation.

## Discussion

Δ^9^-Tetrahydrocannabinol (THC), the primary psychoactive constituent of cannabis, has been widely studied for its immunomodulatory properties (47, 48). Given the availability of new and increasingly potent cannabis-derived products, and the strong biological activity of THC, it is essential to investigate how THC influences inflammatory processes (49, 50). Although evidence suggests that THC can suppress pro-inflammatory cytokines and modulate immune cell function, the precise mechanisms and specific effects remain incompletely understood (51). Therefore, further research is needed to clarify the complex relationship between THC and inflammation.

Our work integrates transcriptomic, cellular, and *in vivo* data to show that THC exerts broad and context-dependent immunomodulatory effects on models of LPS-induced inflammation. In THP-1 monocytic cells, similar to prior reports (52–54), THC independently induced a distinct transcriptional profile characterized by activation of oxidative and redox stress responses, ER stress and unfolded protein response pathways, PPARγ-linked metabolic remodeling, and autophagy-associated genes (**Figure 1**). These processes reflect a shift toward a stress-adapted, metabolically rewired macrophage state. Importantly, under inflammatory conditions, THC broadly suppressed LPS-induced immune activation, including the downregulation of key Type I interferon–stimulated genes (such as CXCL10, CXCL11, APOBEC3G, and APOBEC3A) and immune regulatory molecules such as CD209 and CD274. This indicates broad attenuation of antiviral and antigen-presenting pathways, aligning with prior reports that THC alleviates LPS-induced inflammatory responses (28).

Consistent with its effects in THP-1 cells, THC reduced activation marker expression in primary human monocytes and attenuated splenic myeloid and T-cell activation *in vivo* following LPS challenge. In the CNS, where CB1 signaling predominates (46), THC significantly dampened LPS-induced neuroinflammatory gene expression, reducing expression of inflammatory mediators while promoting genes associated with neurogenesis, circadian regulation, and synaptic plasticity. Together, these findings demonstrate that THC not only modulates peripheral myeloid and T-cell activation but can also reduce neuroinflammatory signaling within the brain.

Myeloid cells are central regulators of inflammation, serving as key effectors of the innate immune response (55). For monocytes and macrophages activation, type I interferons play a key role by driving the upregulation of costimulatory molecules and enhancing antigen presentation, thereby facilitating adaptive immune responses (56, 57). In our study, the downregulation of type I IFN stimulated genes in THC-treated, LPS-stimulated THP-1 monocytic cell aligns with the observed reduction in surface activation markers on primary monocytes. Supporting these data, previous studies have also shown that cannabinoids reduce the expression of type I IFN response genes and activation markers in monocytes (58). By attenuating IFN signaling, THC downregulates activation and costimulatory markers in monocytes, thereby diminishing their responsiveness and antigen-presenting capacity to T cells. This coordinated modulation of ISGs, cytokines and surface activation underscores THC’s ability to regulate innate immune activation at multiple levels.

Heavy cannabis use has been associated with dysregulated immune activation (41). The downregulated expression of activation markers on splenic T-cell and splenic monocytes in mice treated with THC as compared to vehicle shows that chronic exposure to THC may alter both innate and adaptive immune responses. Costiniuk et al., in a randomized trial, also demonstrated that oral cannabinoids reduced systemic inflammation and immune activation markers in people with HIV on ART without affecting viral reservoirs (59). Another study suggested that THC treatment reduces CD8+ T cell mediated activation of astrocytes (60).

Our RNA sequencing analysis of mouse brain tissue revealed that LPS-stimulation elicited a robust upregulation of genes associated with inflammatory and cytokine signaling pathways, consistent with prior reports of LPS-induced (22, 61). In contrast, co-treatment with THC markedly attenuated this response, resulting in the downregulation of inflammation and cytokine-related genes and a concurrent upregulation of genes associated with neurogenesis, circadian rhythm regulation, synaptic plasticity, and modulation of synaptic transmission (62–64). These transcriptional changes suggest that THC mitigates LPS-driven neuroinflammatory responses while promoting pathways linked to neuronal maintenance and homeostasis. Consistent with these findings, previous studies have demonstrated that cannabinoids, including THC, suppress LPS-induced microglial activation through inhibition of ROS- and NF-κB–dependent signaling pathways (65). Cannabinoids and endocannabinoids have been shown to regulate microglial activation, suppress pro-inflammatory cytokine production, and promote a shift toward an anti-inflammatory or neuroprotective phenotype (66, 67). Our study, combining transcriptomic profiling in a human monocytic cell line, primary human monocytes, and *in-vivo* models of endotoxin challenge, build on and significantly expand the existing literature on cannabinoid-mediated immune modulation.

Previous studies using single-cell transcriptomic profiling of human immune cells demonstrated that acute THC exposure (68) and cannabis use (69) induces complex cell type-specific transcriptional changes particularly within monocytes and lymphocyte populations, affecting pathways associated with immune activation, antigen presentation, and inflammation. These findings provide mechanistic support for our observations that THC attenuates LPS-induced inflammatory responses *in vitro* and *in vivo*. In our study, THC reduced LPS-induced monocyte activation markers including CD80, CD83, CD209 and HLA-DR, suggesting that THC exposure affects the activation state and antigen-presenting capacity of monocytes, potentially limiting downstream activation of adaptive immune responses. Consistent with the human single-cell findings showing THC-mediated modulation of lymphocyte-associated transcriptional programs, we observed decreased expression of T-cell activation markers on T cells following THC treatment in LPS challenged mice. Together, these findings suggest that THC alone does not necessarily cause broad immune suppression but rather modulate immune activation by dampening excessive inflammatory signaling. Our findings suggest that THC modulates stress-adaptation and metabolic-remodeling programs and attenuates LPS-induced neuroinflammation. By limiting excessive inflammatory responses, THC may contribute to the modulation of neuroimmune and peripheral immune activation, warranting further investigation into its therapeutic potential in inflammatory and neurodegenerative diseases.

While our study provides a multi-model view of THC-driven immune reprogramming, several limitations should be acknowledged. First, transcriptomic data from THP-1 cells may not fully capture the diversity of primary human monocyte or tissue-resident macrophage responses. Second, the acute THC and LPS exposures used here do not encompass the varied dosing, duration, or co-exposures seen in real-world cannabis use. Finally, the CNS effects observed *in vivo* likely involve multiple cell types and receptors, and the precise mechanisms remain to be defined. Future studies using primary human cells, chronic exposure models, and cell-type–specific approaches will be essential to determine how THC shapes innate immune and neuroimmune states in physiological and disease contexts.

## Methods

### THC treatment of cell lines and primary cells

Δ^9^-Tetrahydrocannabinol (THC) was obtained from the National Institute on Drug Abuse (NIDA) Drug Supply Program as a 20 mg/mL stock solution dissolved in ethanol and stored at –20 °C until use. For *in vitro* experiments, working concentrations of THC were freshly prepared by diluting the stock solution in complete culture medium, ensuring that the final ethanol concentration did not exceed 0.1% (v/v). Human THP-1 monocytic cells were treated with vehicle control (ethanol) or THC at a final concentration of 10 µg/mL and stimulated with or without lipopolysaccharide (LPS; 5 µg/mL; Invivogen, USA). After 8 hours of incubation, cells were harvested for downstream applications including RNA sequencing (RNA-seq) and quantitative real-time PCR. All experiments were independently repeated 3 times. Human peripheral blood mononuclear cells (PBMCs, n=4) were obtained from the UCLA Virology Core. PBMCs were treated with vehicle control (ethanol) or THC at concentrations of 50 ng/mL and 200 ng/mL, in the presence or absence of LPS (5 µg/mL). Following 8 days of stimulation, cells were harvested and stained for flow cytometric analysis of immune activation markers.

### *In vivo* treatment of THC and LPS

Δ^9^-Tetrahydrocannabinol (THC) was obtained from the National Institute on Drug Abuse (NIDA) Drug Supply Program. Wild-type immunocompetent B6/129J mice (male, 200–230 days of age) were randomly assigned to one of the three experimental groups (n=3 mice/group): Mock (vehicle control), LPS only, and THC+LPS. Mice received either a control diet or a THC-supplemented diet (20 mg/kg/day) for 15 consecutive days. A THC-only group was not included because the primary objective of the study was to evaluate the effect of THC on LPS-induced systemic inflammation rather than the effects of THC alone under basal conditions. Beginning on day 12, mice were administered intraperitoneal (I.P.) injections of either vehicle or lipopolysaccharide (LPS; 0.5 mg/kg) once daily for 3 days to induce systemic inflammation. Animals were randomly allocated to treatment groups, and sample collection and downstream, analyses were performed in a blinded manner. Only male mice were used in the study; therefore, no sex-based comparisons were performed. On day 15, mice were euthanized by administration of an overdose of Isoflurane vapor (5%) until complete cessation of respiration and loss of reflexes were confirmed, followed by decapitation as a secondary physical method to ensure death. Decapitation method was performed with the recommendations of the American Veterinary Medical Association (AVMA) Guidelines for the Euthanasia of animals. Tissues including blood, spleen, and brain were collected immediately following euthanasia for downstream analyses such as RNA sequencing and flow cytometry.

### Real-time PCR

To measure the levels of human ISGs (MX1, OAS1, IRF7, IL-1β, TNF-⍺ and HPRT1 as an internal control, THP-1 cells were harvested for RNA extraction and making of cDNA using the High-Capacity cDNA Reverse Transcription Kit (Thermo Fisher Scientific). Real-time PCR was performed using the following human primers and probes:

IL-1β forward primer: TGAGCTCGCCAGTGAAATGA
IL-1β reverse primer: AGATTCGTAGCTGGATGCCG
TNF-⍺ forward primer: GCTGCACTTTGGAGTGATCG
TNF-⍺ reverse primer: TCACTCGGGGTTCGAGAAGA

Single Tube TaqMan Gene Expression Assays (Thermo Fisher Scientific): human HPRT1 (Hs01003267_m1), human MX1 (Hs00895608_m1), human IRF7 (Hs01014809_g1), and human OAS1 (Hs00973635_m1). Relative mRNA expression was calculated by normalizing each gene to housekeeping HPRT1 mRNA expression.

### Flow cytometry

Human primary monocytes were harvested using accutase-enzyme cell detachment medium (Thermo fisher scientific, USA). Following detachment, the cells were stained with the following antibodies: CD45 BV785 (clone HI30), CD80 BV711 (clone 2D10), CD83 PE-Dazzle 594 (clone REA714-HB15), CD209 FITC (clone eB-h209), HLA-DR BV650 (clone L243), CD11b PE-Cy7 (clone ICRF44), CD3 BV605 (clone OKT3), CD4 PE (clone OKT4), CD8a PE-Cy7 (clone RPA-T8), CD38 PE-Cy5 (clone HIT2), CD69 FITC (clone FN50) along with LIVE/DEAD Fixable Yellow Dead Cell Stain Kit (Invitrogen). Flow cytometry analysis was performed using a sequential gating strategy. Lymphocytes and monocytes were initially identified based on forward scatter (FSC) and side scatter (SSC) characteristics. Doublets were excluded using forward scatter area (FSC-A) versus forward scatter width (FSC-W), and singlet cells were retained for further analysis. Cell viability was assessed using Zombie Yellow Fixable Viability Dye, and only Zombie Yellow-negative (live) cells were included in the analysis. From the viable cell population, CD45+ leukocytes were gated. Monocytes were identified as CD45+CD11b+ cells, and the expression of activation markers (CD80, CD83, CD209, and HLA-DR) was analyzed within the CD11b+ population. For lymphocyte analysis, CD3+CD45+ T cells were gated from the CD45+ population, followed by identification of CD4+ and CD8+ T-cell subsets. The expression of activation markers (CD38, CD69, and HLA-DR) was subsequently evaluated within both CD4+ and CD8+ T-cell populations (**Supplementary Figure 2**).

For mouse studies, single-cell suspensions were prepared from spleen. Splenocytes were stained with the following antibodies: CD45 BV785 (clone 30-F11), CD3 AF700 (clone 17A2), CD11b APC-eFluor 780 (clone M1/70), CD19 BV711 (clone 6D5), CD38 APC (clone 90), CD69 PE-Cy5 (clone H1.2F3) and CD80 BV421 (clone 16-10A1). LIVE/DEAD Fixable Yellow Dead Cell Stain Kit (Invitrogen) was used. A similar sequential gating strategy was applied. After exclusion of doublets and dead cells, CD45+leukocytes were selected. Monocytes were identified as CD45+CD11b+ cells, and CD80 expression was evaluated within this population. For lymphocyte analysis, CD3+CD19- T cells were gated from the CD45+ population. The expression of activation markers (CD38 and CD69) was subsequently evaluated from CD3+CD19- T-cell populations (**Supplementary Figure 3**). Single-color controls were used to establish compensation matrices for multicolor flow cytometric analysis. All staining incubations were performed for 20 min at 4 °C in the dark. The cells were acquired using an LSRFortessa flow cytometer and FACSDiva software (BD Biosciences, USA). Data were analyzed using FlowJo version 10.10 software (BD Biosciences, USA).

### THC quantification in plasma by HPLC

Plasma samples collected at necropsy were used to quantify THC levels using high-performance liquid chromatography (HPLC). Samples were processed as follows: 1 mL of acetonitrile containing formic acid was added to each plasma sample to precipitate proteins and extract cannabinoid. The mixtures were vortexed briefly and centrifuged at 16,000 × g for 5 minutes to pellet any precipitated material. Solid-phase extraction (SPE) was carried out using Phree™ SPE cartridges (Phenomenex). The SPE apparatus was assembled using glass test tubes and 1.5 mL microcentrifuge tubes. The clarified supernatants were carefully transferred to the Phree cartridges and eluted by centrifugation at 500 × g for 2 minutes. The eluates were collected into clean microcentrifuge tubes and retrieved using sterile tweezers. Eluted samples were dried using a vacuum centrifuge, then resuspended in 30 µL of methanol:water (70:30, v/v). A 20 µL aliquot of each resuspended sample was injected into the HPLC system and analyzed using an LTQ-XL linear ion trap mass spectrometer (Thermo Scientific, USA).

### RNA-seq

Total RNA was extracted from both human THP-1 cells and mouse brain samples. Human THP-1 monocytic cells were treated with vehicle (ethanol) or THC (10 µg/mL), in the absence or presence of LPS (5 µg/mL) for 8 hours. Whole mouse brain tissues were collected from animals fed either a control diet or a THC-supplemented diet, with or without LPS injection. RNA extraction was performed using RNeasy Kits for RNA isolation and purification (Qiagen, USA), according to the manufacturer’s protocol. Stranded mRNA libraries were prepared using poly-A selection following the standard Illumina Stranded mRNA Library Prep protocol, and sequencing was performed on an Illumina NovaSeq X Plus 10B platform, generating paired-end reads at 100 million reads per sample.

RNA-seq data processing was performed separately for each dataset. The mouse dataset included nine samples (n=9, three mice per group), whereas the THP-1 dataset included 12 samples (n=12, three independent biological replicates per group). Initial quality assessment was performed with FastQC (v0.11.9 for human; v0.12.1 for mouse). Adapter sequences and low-quality bases were trimmed using Trim Galore! (v0.6.7) with Cutadapt (v3.4). Human reads were aligned to the GRCh38 reference genome using STAR (v2.7.10a), while mouse reads were independently aligned to the GRCm38 reference genome (Ensembl annotation) using STAR (v2.7.10a). Transcript-level quantification was performed using Salmon (v1.10.1) in alignment-based mode. Aligned reads were sorted and indexed using SAMtools (v1.16.1 for human; v1.2 for mouse), and duplicate reads were marked using Picard MarkDuplicates (v3.0.0 for human; v3.1.1 for mouse). Transcript assembly was performed with StringTie (v2.2.1), and coverage tracks in bigWig format were generated using BEDTools (v2.30.0 for human; v2.31.1 for mouse) and UCSC bedGraphToBigWig. Comprehensive quality control metrics were evaluated using RSeQC (v3.0.1 for human; v5.0.2 for mouse), Qualimap (v2.2.2 for human; v2.3 for mouse), and dupRadar (v1.28.0 for human; v1.32.0 for mouse). Gene-level count matrices were generated via tximeta/tximport, and differential expression analysis was performed using DESeq2 (v1.28.0) (adjusted p < 0.05, |log_2_FC| > 1). Downstream functional analyses, including Gene Ontology (adjusted p < 0.1) and Reactome pathway enrichment (adjusted p < 0.1), were conducted using clusterProfiler in R. The RNA-seq data is deposited in GEO, GSE328034.

### Statistical analysis

The results are presented as Mean ± S.D. Statistical analysis was performed using GraphPad Prism v10.5.0 software (GraphPad Software Inc., San Diego, CA, USA). All experiments using THP-1 cells were carried out 3–5 independent times unless otherwise stated. Experiments with primary monocytes were carried out independently using PBMCs from 4 healthy donors. Mice experiments were conducted with 3–4 animals per group. To compare statistical differences between 2 groups, the data were analyzed using Mann-Whitney U tests to evaluate the differences between experimental groups. When multiple comparisons were performed, the Bonferroni-Dunn *post hoc* correction was applied to maintain the family-wise error rate at 5%. For RNA-seq data, differential expression analysis was performed using the Wald test, and P values were adjusted for multiple testing using the Benjamini–Hochberg false discovery rate (FDR) procedure; genes with an adjusted P value < 0.05 were considered differentially expressed. P values less than 0.05 was considered significant (*P < 0.05, **P < 0.005, ****P < 0.0005).

## Data Availability

The datasets presented in this study can be found in online repositories. The names of the repository/repositories and accession number(s) can be found below: GSE328034 (GEO).
