# Supplementary material for: Delta-9-tetrahydrocannabinol alleviates inflammation by modulating immune responses in LPS-induced murine inflammation model
Source: Front Immunol. 2026 Sep 16;17:1856131. doi: 10.3389/fimmu.2026.1856131 (PMC13623581; doi:10.3389/fimmu.2026.1856131)
Supplement: Supplementary file 1 [file DataSheet1.pdf]

**Supplementary Figure 1**

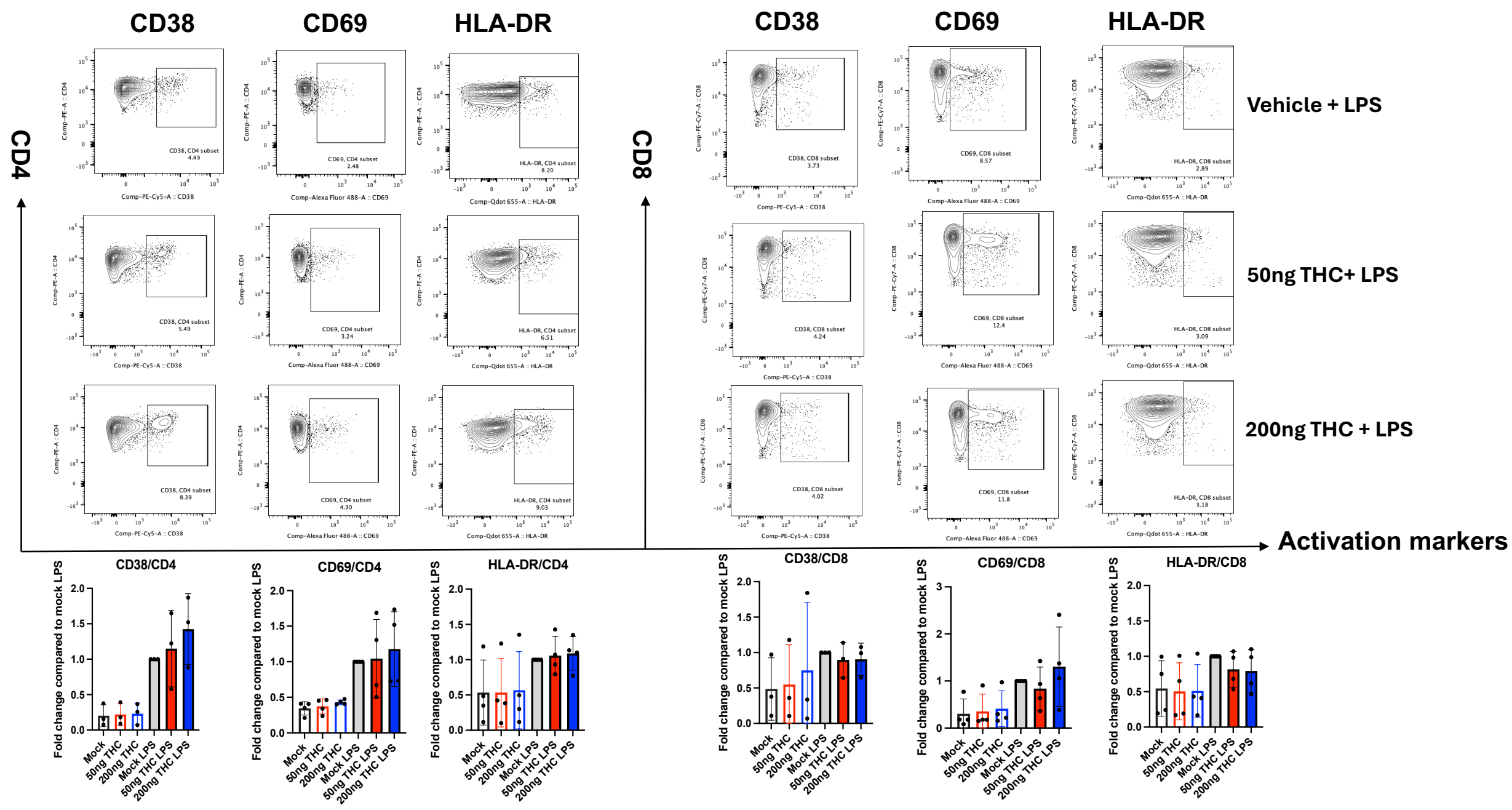


**Supplementary Figure 1:** THC does not affect the expression of activation markers in primary T cells: CD14- cells were treated with vehicle or THC (50ng/ml, 200ng/ml) in the presence and absence of LPS (5ug/ml) for 8 days. Cells were harvested and stained for activation markers CD38, CD69 and HLA-DR. (A) Representative flow plots for activation markers gated on CD4+ and CD8+ T-cells. (B) Summary of fold change compared to mock and LPS in terms of percentage of cells positive for activation markers from 4 healthy donors.

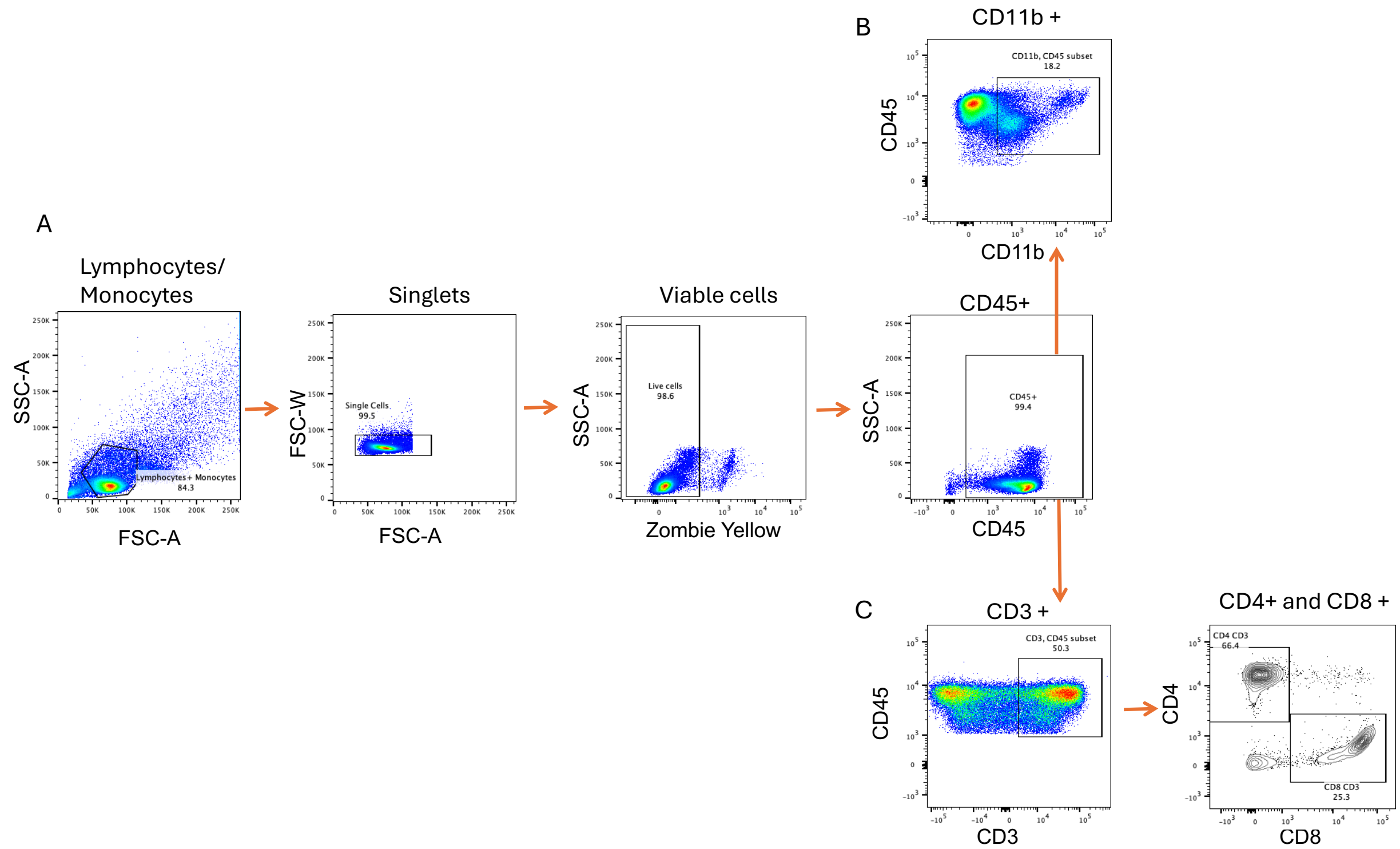


**Supplementary Figure 2: Gating strategies for flow cytometric analysis of PBMCs.** (A) Representative gating strategy for identification of lymphocytes and monocytes. Singlets and viable cells were first selected, followed by gating on $CD45^+$ leukocytes. (B) Gating strategy for monocytic cells: $CD11b^+$ cells were gated from the $CD45^+$ population. The $CD11b^+CD45^+$ double-positive population was subsequently used to assess activation markers, including CD83, CD80, CD209, and HLA-DR. (C) Gating strategy for T cells: $CD3^+$ cells were gated from the $CD45^+$ population. The $CD3^+CD45^+$ double-positive population was further subdivided into $CD4^+$ and $CD8^+$ T cell subsets. Activation markers, including CD38, CD69, and HLA-DR, were analyzed within both $CD4^+$ and $CD8^+$ populations.

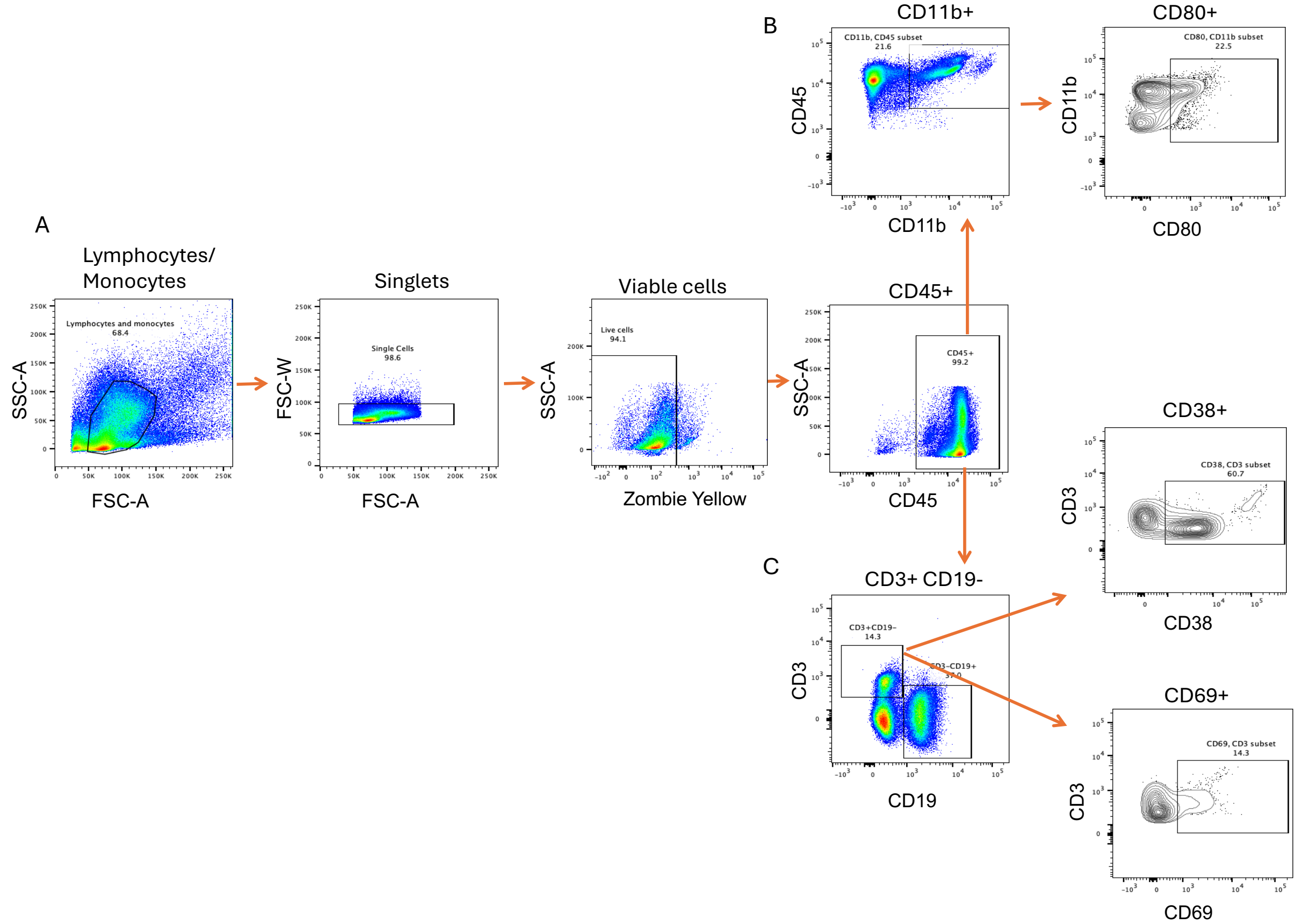


**Supplementary Figure 3: Gating strategies for flow cytometric analysis of mouse splenocytes.** (A) Representative gating strategy for identification of mouse lymphocytes and monocytes. Singlets and viable cells were first selected, followed by gating on $CD45^+$ leukocytes. (B) Gating strategy for monocytic cells: $CD11b^+$ cells were gated from the $CD45^+$ population. The $CD11b^+CD45^+$ double-positive population was subsequently used to assess activation marker CD80 (C) Gating strategy for lymphocytes: $CD3^+CD19^-$ cells were gated from the $CD45^+$ population. Activation markers, including CD38 and CD69 were analyzed within $CD3^+CD19^-$ populations. The representative figure is from LPS treated mice.

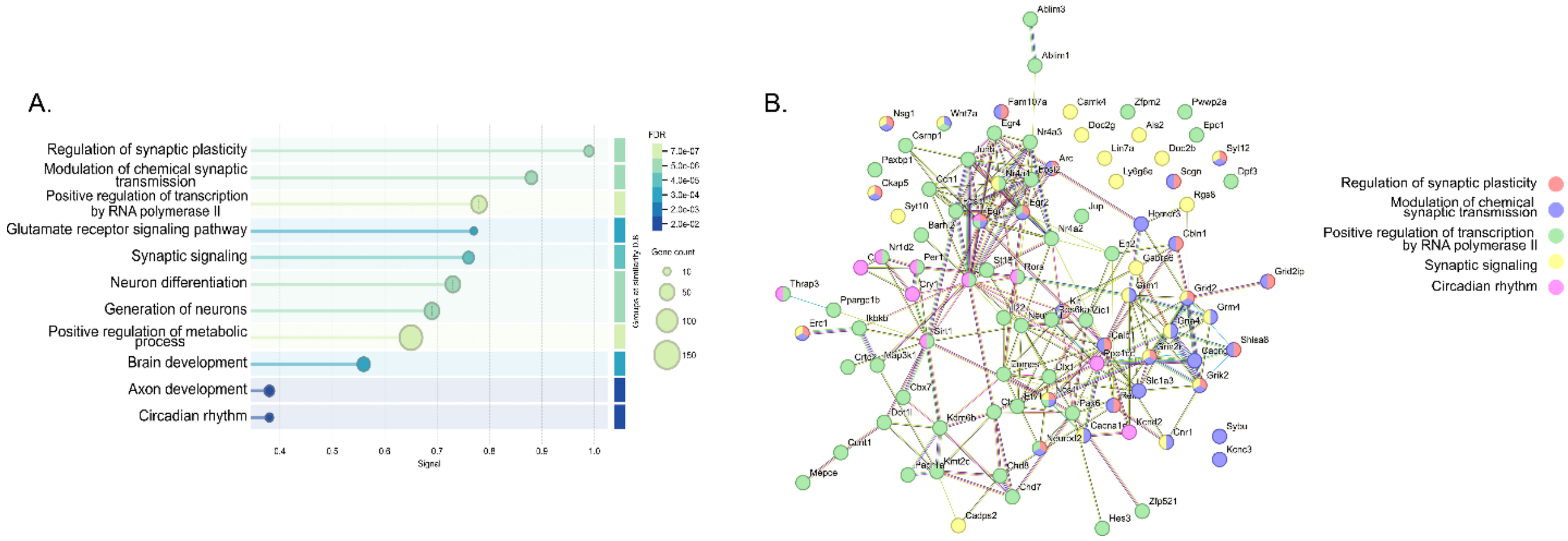


**Supplementary Figure 4. Gene Ontology enrichment and protein interaction network analysis of upregulated genes in LPS+THC vs. LPS comparison.** (A) Dot plot showing significantly enriched Gene Ontology (GO) biological processes among upregulated genes. The x-axis represents signal strength; circle size reflects gene count within each term; color indicates false discovery rate (FDR), with light green corresponding to greater statistical significance. Enriched processes include regulation of synaptic plasticity, modulation of chemical synaptic transmission, positive regulation of transcription by RNA polymerase II, glutamate receptor signaling pathway, synaptic signaling, neuron differentiation, generation of neurons, brain development, axon development, and circadian rhythm. (B) STRING protein–protein interaction network of upregulated genes. Each node represents a gene, and edges reflect known or predicted functional interactions. Node color indicates GO category membership. Nodes participating in multiple pathways are shown with split coloring.
