# Supplementary material for: Delta-9-tetrahydrocannabinol alleviates inflammation by modulating immune responses in LPS-induced murine inflammation model
Source: Front Immunol. 2026 Sep 16;17:1856131. doi: 10.3389/fimmu.2026.1856131 (PMC13623581; doi:10.3389/fimmu.2026.1856131)
Supplement: Supplementary file 2 [file DataSheet2.pdf]

frontiers

The International Society for the Advancement of Cytometry (ISAC) has highlighted the importance of including comprehensive methodological information to ensure data reproducibility and reliability. In line with this, Frontiers in Immunology now requires authors to submit a checklist for manuscripts that involve flow or mass cytometry. This checklist helps standardize the reporting process, improving the quality and transparency of published data. By doing so, we support scientific progress, making it easier for other researchers to replicate and validate experiments.

This form should be submitted with any manuscripts using flow or mass cytometry.

Sample/specimen/material description

☐ Total blood

☑ PBMCs

☑ Organ digests

Other Splenocytes

Did the samples suffer any treatment before or after incubation with the antibodies?

☐ Drug ____

☐ Cell permeabilization ____

☐ Dye ____

☐ Propidium iodine

☐ Not applicable

Other Fixation (PBS+1% formaldehyde)

Instrument and antibodies

Name of the Cytometer BD LSR Fortessa

Antibodies and targets	Fluorochrome/ Metal	Catalog number/Company
e.g. anti-CD4	FITC	Cat. XXX/ XXX ltd.
anti-human CD45	BV785	304048/ Biolegend

anti-human CD11b	PE-Cy7	25-0118-42/ Invitrogen
anti-human CD80	BV711	305236/ Biolegend
anti-human CD83	PE/Dazzle 594	305328/ Biolegend
anti-human CD209	FITC	11-2099-42/ Invitrogen
anti-human HLA-DR	BV650	307650/ Biolegend
anti-human CD3	BV605	317322/ Biolegend
anti-human CD4	PE	317410/ Biolegend
anti-human CD8a	PE-Cy7	25-0088-42/ Invitrogen
anti-human CD38	PE-Cy5	15-0389-42/ Invitrogen
anti-human CD69	FITC	310904/ Biolegend
anti-mouse CD45	BV785	103149/ Biolegend
anti-mouse CD11b	APC-eFluor 780	101226/ Biolegend
anti-mouse CD80	BV421	104725/ Biolegend
anti-mouse CD3	AF700	100216/ Biolegend
anti-mouse CD38	APC	102712/ Biolegend
anti-mouse CD69	PE-Cy5	104509/ Biolegend
anti-mouse CD19	BV711	115555/ Biolegend
Live/dead Fixable Yellow	Yellow (405 excitation)	L34959/ Invitrogen

Data analyses

1. Name of the software FlowJo 10.10.0 (BD)_________________________________

2. Reference gating strategy in the manuscript or supplementary material

Gating strategy in (eg Figure X) Supplementary figure 2 and 3_______________________________
