# Supplementary material for: Delta-9-tetrahydrocannabinol alleviates inflammation by modulating immune responses in LPS-induced murine inflammation model
Source: Front Immunol. 2026 Sep 16;17:1856131. doi: 10.3389/fimmu.2026.1856131 (PMC13623581; doi:10.3389/fimmu.2026.1856131)
Supplement: Supplementary file 3 [file SupplementaryFile1.pdf]

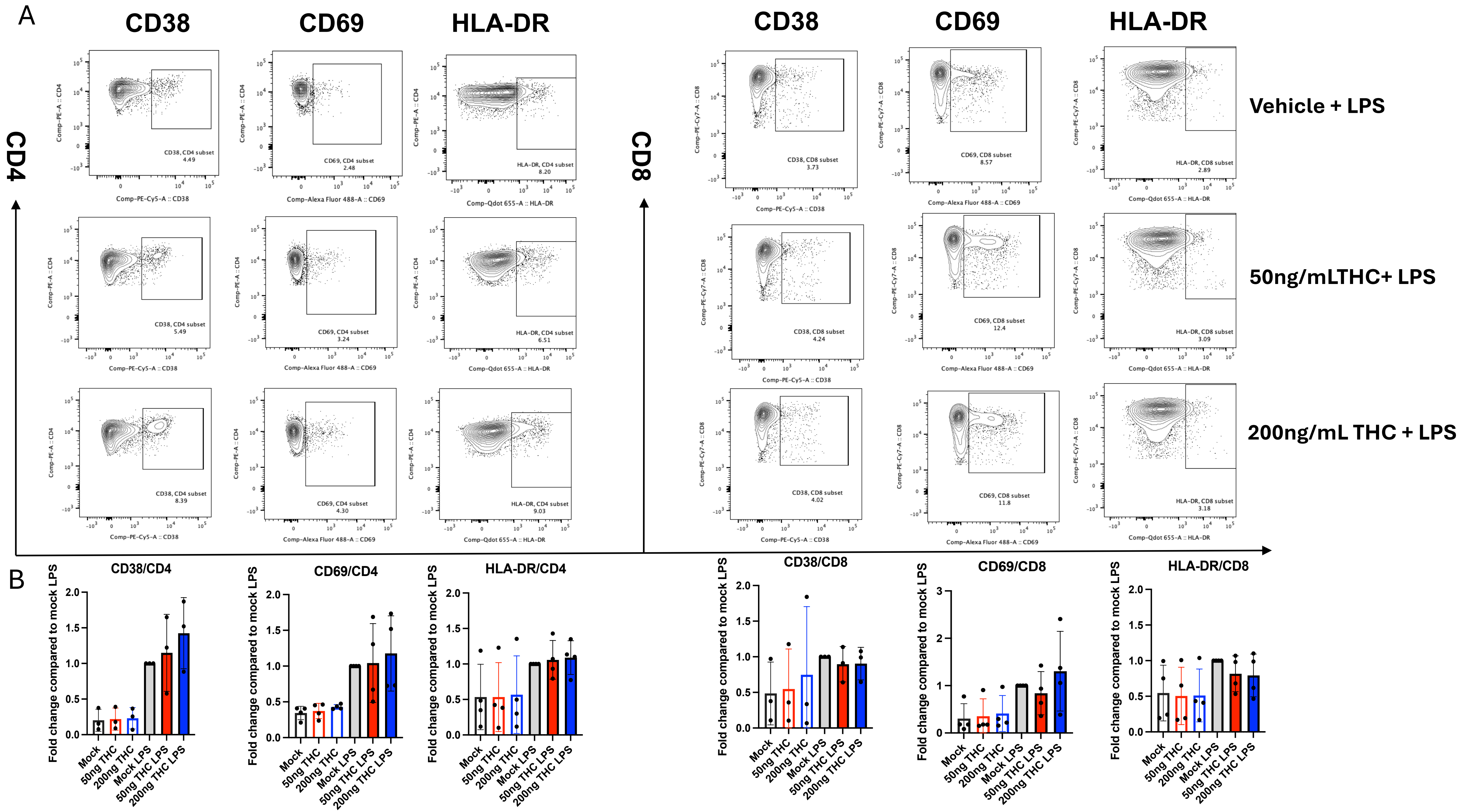

Supplementary Figure 1: THC does not affect the expression of activation markers in primary T cells: PBMCs were treated with mock (ethanol) and THC (50ng/mL, 200ng/mL) in the presence and absence of LPS (5ug/mL) for 8 days. Cells were harvested and stained for activation markers CD38, CD69 and HLA-DR. (A) Representative flow plots for activation markers, (B) Summary of fold change compared to mock and LPS in terms of percentage of cells positive for activation markers from 4 healthy donors.
